# Differential Pharmacological Activities of Oxygen Numbers on the Sulfoxide Moiety of Wasabi Compound 6-(Methylsulfinyl) Hexyl Isothiocyanate in Human Oral Cancer Cells

**DOI:** 10.3390/molecules23102427

**Published:** 2018-09-21

**Authors:** Min-Ju Lee, Wen-Ser Tseng, Jerry Cheng-Yen Lai, Hui-Ru Shieh, Chih-Wen Chi, Yu-Jen Chen

**Affiliations:** 1 Taipei First Girls High School, Taipei 10045, Taiwan; minrulee0908@gmail.com; 2 Department of Medical Research, MacKay Memorial Hospital, New Taipei City 25160, Taiwan; selene0508@hotmail.com (W.-S.T.); ru123@mmh.org.tw (H.-R.S.); 3 Department of Medical Research, Taitung MacKay Memorial Hospital, Taitung City 95054, Taiwan; chengyen@hotmail.com; 4 Department of Nursing, MacKay Medical College, New Taipei City 25245, Taiwan; 5 Department of Medical Research, China Medical University Hospital, Taichung 40402, Taiwan; 6 Department of Radiation Oncology, MacKay Memorial Hospital, Taipei 10449, Taiwan

**Keywords:** wasabi, 6-MITC, I7447, I7557, oral cancer, mitosis, apoptosis

## Abstract

6-(methylsulfinyl) hexyl isothiocyanate (6-MITC) is a naturally occurring compound isolated from *Wasabia japonica* (wasabi). The synthetic derivatives, 6-(methylsulfenyl) hexyl isothiocyanate (I7447) and 6-(methylsulfonyl) hexyl isothiocyanate (I7557), were derived from 6-MITC with the deletion and addition of oxygen, respectively. We aimed to evaluate the effect of these synthetic compounds on human oral cancer cells, SAS and OECM-1. All three compounds (I7447, 6-MITC, and I7557) inhibited the viability of SAS and OECM-1 cells using MTT assay. Morphological observations showed various proportions of mitotic arrest and apoptosis in cells treated with these compounds. Cell cycle analysis revealed relatively abundant G2/M arrest in 6-MITC and I7557-treated cells, whereas sub-G1 accumulation was found in I7447-treated cells. In using phosphorylated histone H3 as a marker for mitosis, the addition of 6-MITC and I7557 (excluding I7447) could be shown to arrest cells during mitosis. In contrast, I7447 induced more prominent apoptosis than the 6-MITC or I7557 compounds. The down-regulated expression of the phosphorylated form of CHK1 and Cdc25c was noted in 6-MITC and I7557-treated cells. I7557 could sensitize SAS cells to death by radiation. The wasabi compound, 6-MITC, and its chemical derivatives with different numbers of oxygen may have differential pharmacological effects on human oral cancer cells.

## 1. Introduction

**Figure 1:**
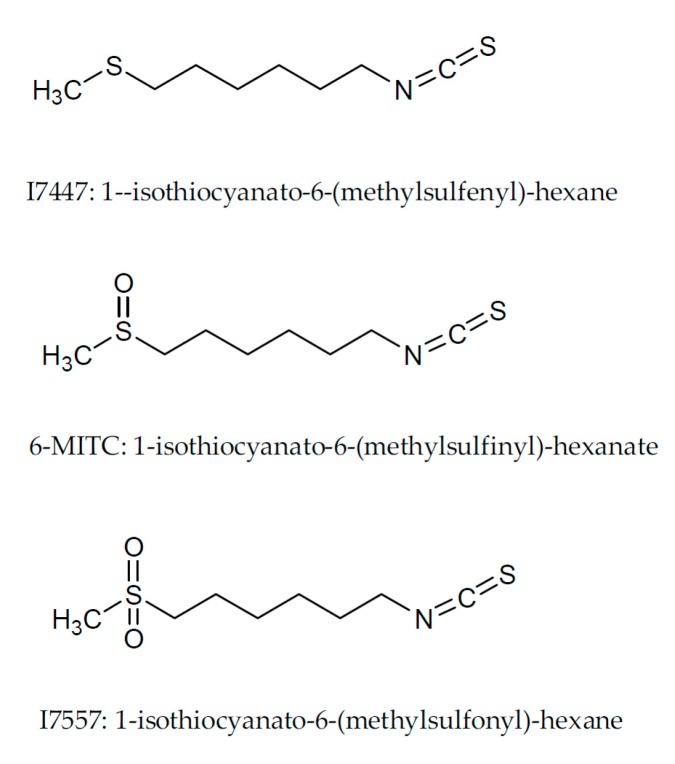
Chemical structures of 6-MITC and its derivatives, I7447 and I7557.

The naturally occurring compound, 6-(methylsulfinyl) hexyl isothiocyanate (6-MITC), isolated from pungent spice, *Wasabia japonica* (wasabi), has been reported to have anti-metastasis [1], chemopreventive [2], and anti-cancer [3] effects. Chemical modification of its sulfone moiety with the deletion and addition of oxygen molecules has yielded 6-(methylsulfenyl) hexyl isothiocyanate (I7447) and 6-(methylsulfonyl) hexyl isothiocyanate (I7557), respectively, as shown in Figure 1.

Oral cancer is the most common malignancy of head and neck cancer. The global incidence of oral cancer is 3.8% and is expected to increase gradually over the next two decades [4]. Being highly invasive and metastatic, squamous cell carcinoma is the most common histology diagnosed in patients with oral cancers. While surgery remains standard care for most patients [5], several modalities including radiation therapy (RT), chemotherapy, and targeted therapy are also used in the treatment of patients with oral cancer [6]. However, there is still a high recurrence rate of up to 30–50%, even after the receipt of post-operative, adjuvant radiotherapy or chemotherapy for oral cancer [7,8]. In clinical practice, the development of novel, effective therapeutics against oral cancer is critically important.

Patients with locally-advanced diseases, unresectable tumors, or patients at high risk, post-operation, are often treated with RT [9]. The concurrent administration of chemotherapy during the RT course has been reported to significantly improve overall, disease-specific, and disease-free survival rates [10,11]. Although the toxicity of combination modality therapy remains higher than that of chemotherapy, RT alone can also lead to significant oral complications and profoundly influence patients’ quality of life [12]. Radiosensitizers are agents that sensitize cancer cells, making them more vulnerable to radiotherapy while sparing surrounding normal tissues. Oxygen has a strong affinity for electrons and can sensitize hypoxic tumor cells to radiation [13]. Therefore, there is an on-going clinical incentive to develop novel radiosensitizers with lower chemotoxicity or to lower the dosage of radiation. Several compounds have been reported to enhance the radiosensitivity of oral cancer cells [14,15,16]. The wasabi compound, 6-MITC, has been reported to enhance the sensitivity of tumor cells to the cell-growth-inhibition effects of anticancer agents [17].

In this study, we examine the differential effects of wasabi compound, 6-MITC, and its chemical derivatives on human, oral squamous, and carcinoma cells, including the effects of radiosensitizing.

## 2. Results

### 2.1. Wasabi Compound and Its Derivatives Inhibited Growth of Oral Cancer Cells

**Figure 2:**
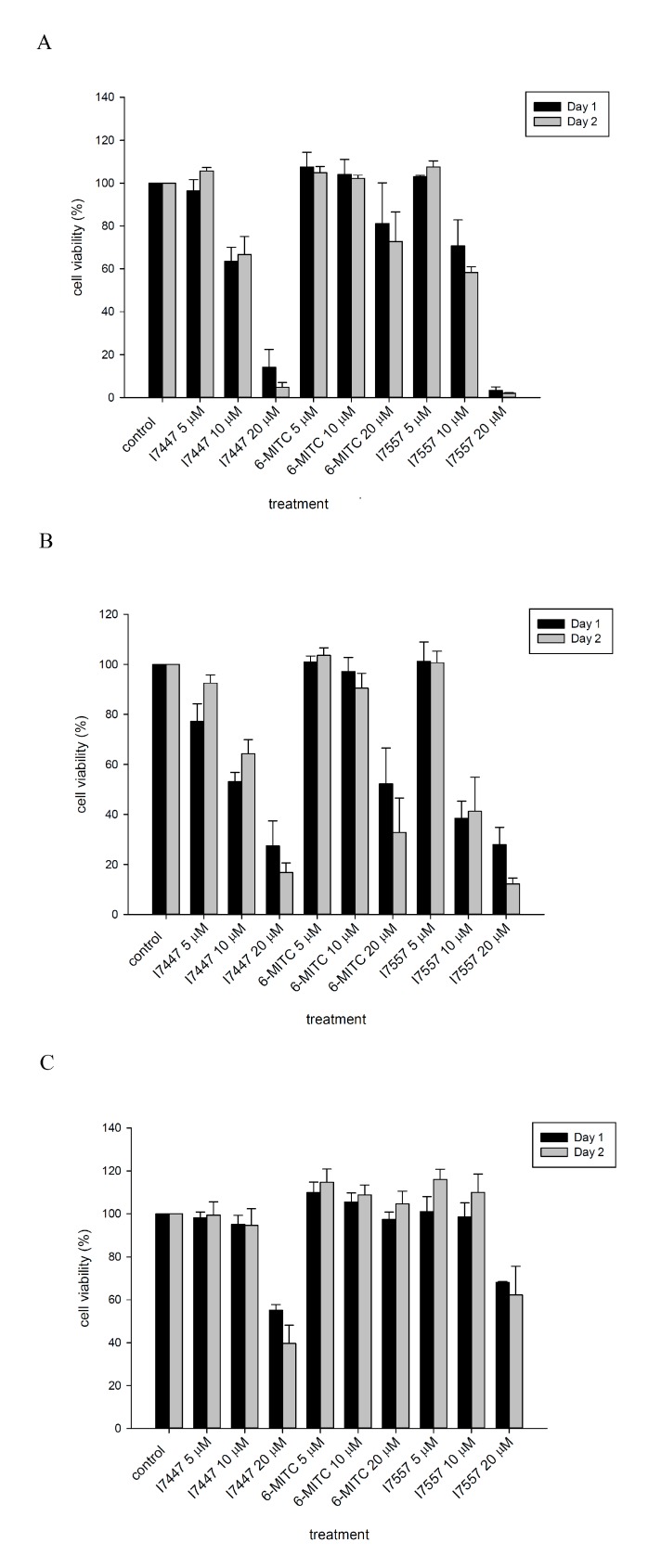
Cell viability of human oral cancer and normal human fibroblast (HFW) cells. The human oral squamous cells, carcinoma SAS (**A**) and OECM-1 (**B**), and HFW cells (**C**) were treated with 6-MITC, I7447, or I7557 for 24 and 48 h. Cell viability was measured using MTT assay. Data from three separate experiments were expressed as the mean ± SEM.

The naturally occurring wasabi compound, 6-MITC, as well as its synthetic derivatives I7447 and I7557, inhibited the viability of oral cancer, SAS, and OECM-1 cells in a dose-dependent manner, as shown in Figure 2A,B. Cell growth inhibition was much less in 6-MITC-treated cells compared to cells treated with its synthetic derivatives. The IC_50_ values for I7447 and I7557 were 10.3 and 10.1 µM in SAS cells, and 13.1 and 9.6 µM in OECM-1 cells, respectively, after 48 h of treatment. At high concentrations of up to 20 µM, both synthetic derivatives revealed moderate toxicity in normal, human fibroblast (HFW) cells, as shown in Figure 2C. Wasabi compounds had less cytotoxicity compared to normal HFW cells.

### 2.2. Morphology Examination Exhibited Mitotic Arrest and Apoptosis after Treatment of Three Compounds

**Figure 3:**
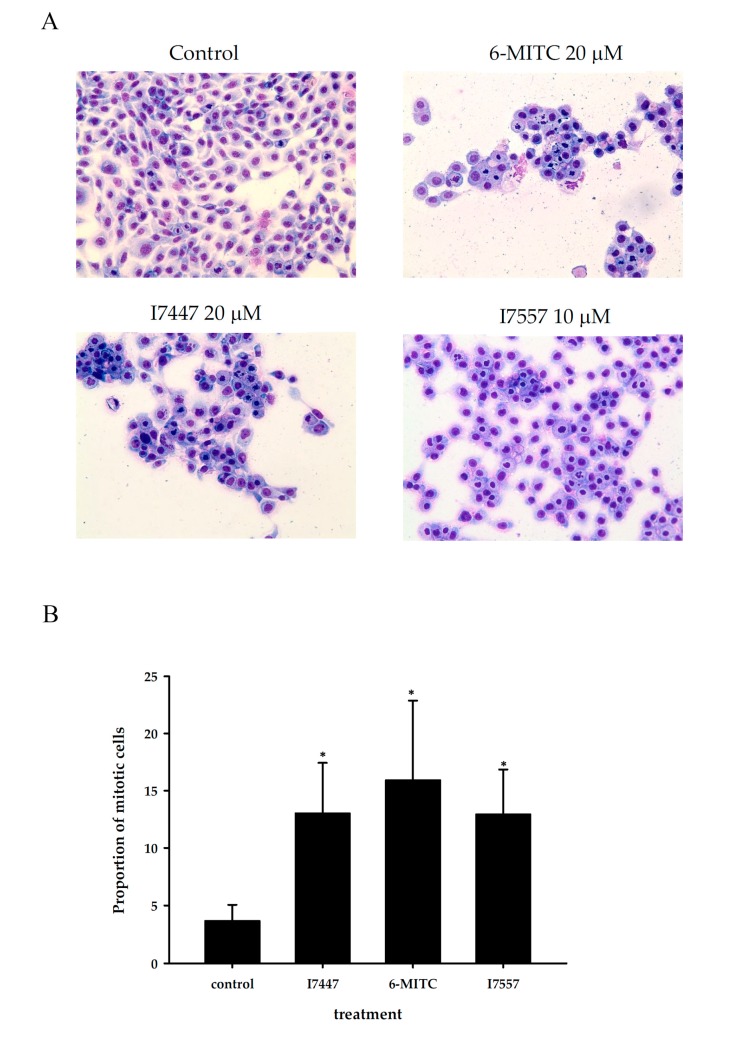
Morphology of oral cancer SAS cells treated with wasabi compound, 6-MITC, and its derivatives I7447 and I7557. SAS cells were exposed to 6-MITC, I7447, and I7557 for 24 h; their morphologies were stained with Liu’s stain and photographed under light microscopy at a magnitude of 400-fold (**A**). The proportion of mitotic cells induced by the wasabi compound and their synthetic derivatives were quantified (**B**). Significant changes between control cells and cells treated with I7447, 6-MITC, and I7557 are indicated by an asterisk, *p* < 0.05.

As shown in Figure 3, morphological observations revealed marked mitotic arrest and apoptosis in cells treated with these three compounds 6-MITC, I7447 and I7557, with each showing different proportions of mitosis and apoptosis. The proportion of mitotically-arrested cells induced by the three compounds increased from 3.8% in the control to 16.0%, 12.4%, and 13.5% in treatments using 20 µM of 6-MITC, 10 µM of I7557, and 20 µM of I7447, respectively.

### 2.3. Wasabi Compound and Its Derivatives Induced G2/M Phase Arrest and Apoptosis in Oral Cancer Cells

**Figure 4:**
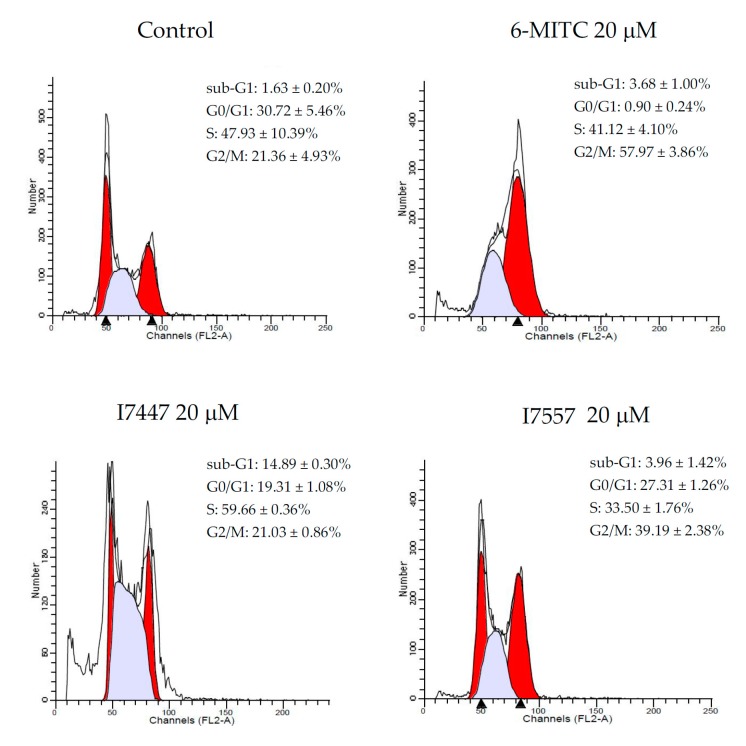
Cell cycle analysis of SAS cells treated with wasabi compound, 6-MITC, and its derivatives I7447 and I7557. Cells were treated with 6-MITC, I7447, and I7557 at 20 µM for 24 h. After treatment, cells were fixed and stained with propidium iodide. Cell cycle distribution was acquired by flow cytometry. Representative DNA histograms of SAS cells, treated with the three compounds, were shown, and the expression level of each cell cycle phase was indicated in the panels. Data from the three separate experiments were expressed as the mean ± SEM.

Cell cycle analysis revealed an increase in the percentage of the G2/M phase in SAS cells, from 21.36 ± 4.93% in the control to 57.97 ± 3.86% in the 6-MITC treatment, 21.03 ± 0.86% in the I7447 treatment, and 39.19 ± 2.38% in the I7557 treatment, as seen in Figure 4. The percentage of apoptotic cells estimated by the hypoploid population was most prominent in those treated with I7447. Apoptotic cells elevated 9.1-fold after exposure to 20 µM of I7447 treatment, when compared to the control cells. The results suggest that 6-MITC, I7557, and I7447 induced SAS cell death through different pathways.

### 2.4. 6-MITC and I7557 Induced Expression of Phosphorylated Histone H3

**Figure 5:**
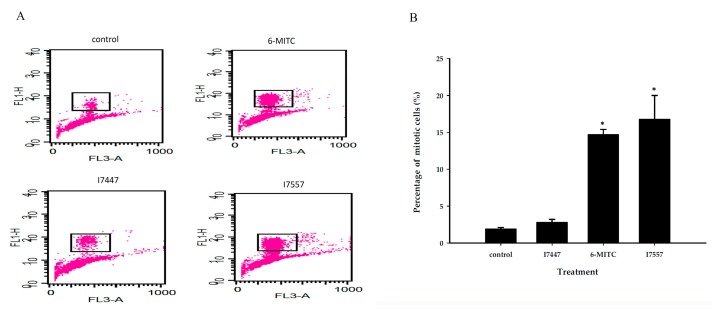
Phosphorylated histone H3 analysis of SAS cells treated with wasabi compound, 6-MITC, and its derivatives I7447 and I7557. After oral cancer SAS cells were treated with 6-MITC, I7447, and I7557 at 20 µM for 6 h, phosphorylated histone H3 expression was detected by flow cytometry. Representative histograms of phosphorylated histone H3 expression are shown (**A**). The amounts of phosphorylated histone H3 were analyzed from three independent experiments using CellQuest^Pro^ software (**B**). Significance between control cells and cells treated with 6-MITC and I7557 is indicated by an asterisk, *p* < 0.05.

To distinguish further the cell cycle arrest at G2/M more specifically, we used phosphorylated histone H3 as a marker for mitosis. Figure 5 shows that 6-MITC and I7557 caused more prominent accumulation of the M phase. I7447 treatment resulted in less M phase, along with a greater hypoploid population, indicative of the development of apoptosis.

### 2.5. Wasabi Compound and Its Derivatives Down-Regulated Expression of Phosphorylated CHK1, Cyclin B1, and Cdc25c

**Figure 6:**
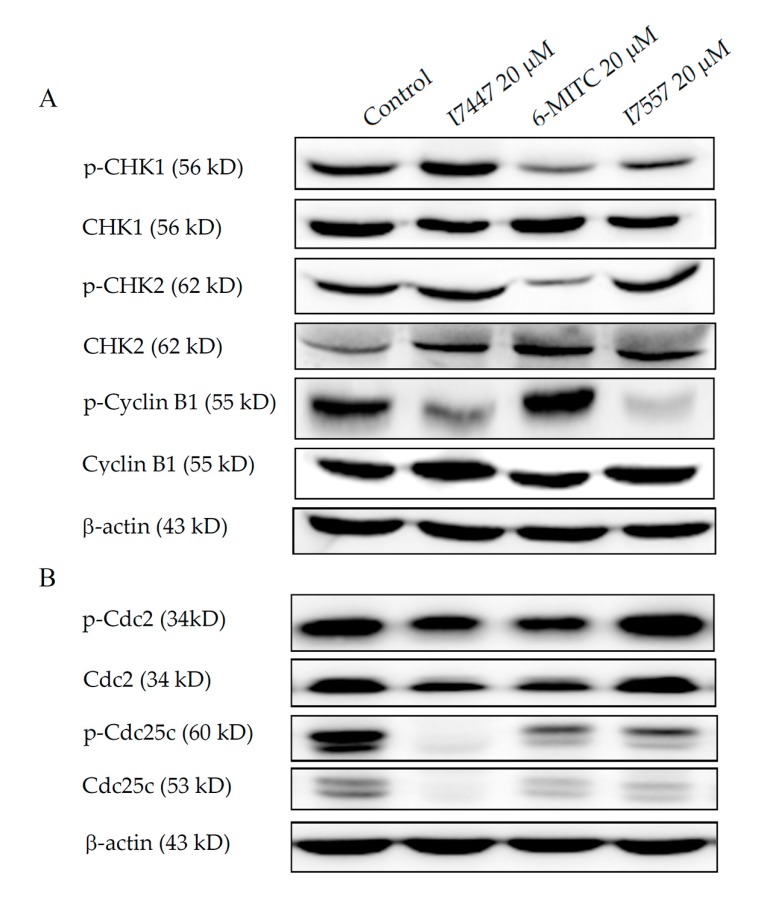

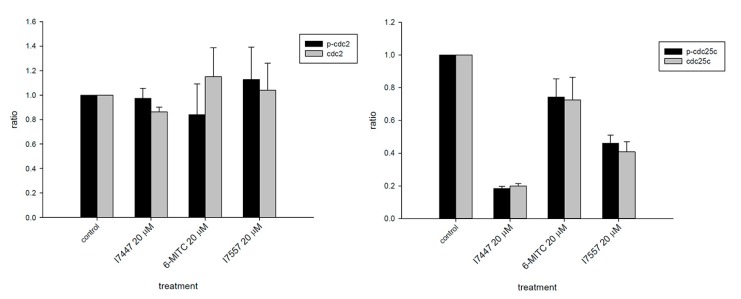
Expression of mitosis-related signaling molecules in SAS cells. Cells were treated with 20 µM of 6-MITC, I7447, and I7557 for 6 h, and then cell lysates were harvested. Equal amounts of proteins were subjected to western blot to detect mitosis-related protein expression. The representative immunoblots of mitosis-related proteins were indicated (**A**,**B**). The bottom part (**B**) shows levels of non-phosphorylated and phosphorylated Cdc2 and Cdc25c, relative to cells treated with vehicle alone (**B**).

After being treated with 6-MITC and I7557, but not I7447, the expression of the phosphorylated form of CHK1 was down-regulated in SAS cells. The expression of phosphorylated cyclin B1 was suppressed by I7447 and I7557, as shown in Figure 6A. All three compounds down-regulated the expression levels of phosphorylated and non-phosphorylated forms of Cdc25c, but not Cdc2, as shown in Figure 6B.

### 2.6. 6-MITC and I7557 Sensitized Oral Cancer Cells to Radiation

**Figure 7:**
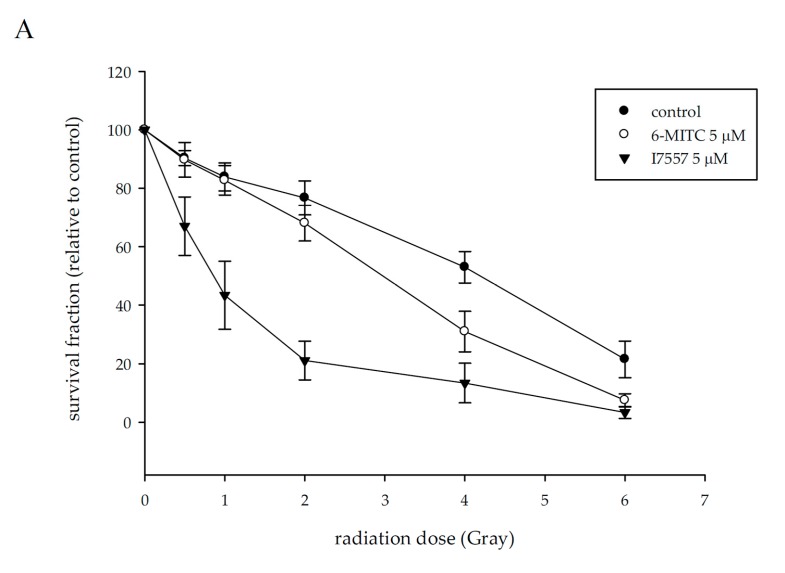

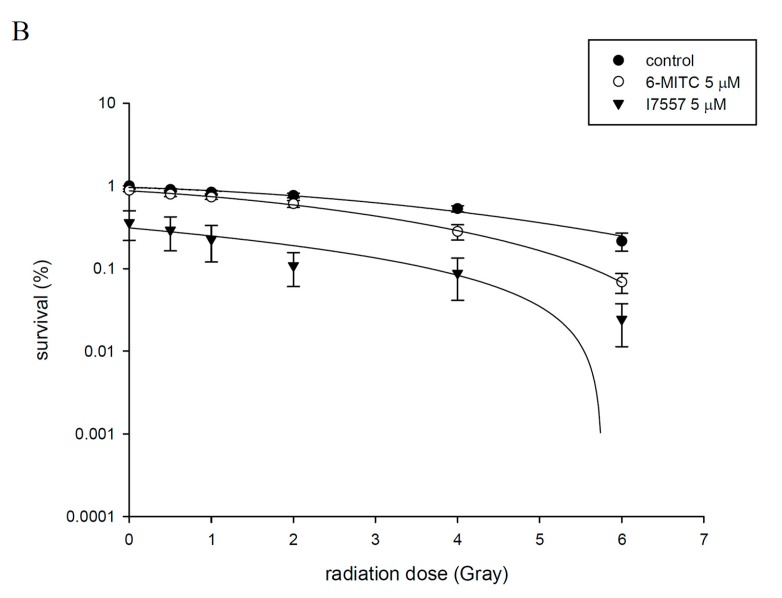
The effect of wasabi compound of 6-MITC and its derivative, I7557, on radiosensitivity of SAS cells. Cells were treated with 5 µM of 6-MITC, I7447, and I7557 for 24 h before irradiation. After irradiation, drugs were washed out, and cells cultured for 8 days in colony formation assay. The survival fraction of radiation, combined with 6-MITC or I7557, was found (**A**). The SER of 6-MITC and I7557 was calculated using a linear-quadratic model (**B**). Results from five separate experiments are expressed as the mean ± SEM.

Radiation reduced the surviving fraction of SAS cells to 90.3 ± 2.6, 83.6 ± 4.8, 76.4 ± 5.7, 52.2 ± 3.0, and 21.1 ± 5.9% at a dose of 0.5–6 Gy. Pretreatment with 6-MITC and I7557 at 5 µM markedly decreased the survival of irradiated tumor cells, as shown in Figure 7A. I7557 possessed greater radiosensitizing activity than 6-MITC against SAS cells, as depicted by the higher sensitizer enhancement ratios (SERs) of I7557 to 6-MITC [SER: I7557 (4.64) versus 6-MITC (1.02)], as shown in Figure 7B. I7447 inhibited colony formation of SAS to less than 50%, which was not suitable for being tested for radiosensitization.

## 3. Discussion

The naturally occurring, bioactive compound, 6-MITC, derived from wasabi, as well as its chemical derivatives I7447 and I7557, were demonstrated to have differential effects on human oral cancer cells in this study. The structure-activity relationship of these three compounds revealed different degrees of anti-tumor activity. I7447 (sulfide containing no oxygen) and I7557 (sulfone containing 2 oxygens) had greater inhibition effects than the naturally occurring compound, 6-MITC (sulfoxide containing 1 oxygen). This indicated that the extent of anti-tumor effects might be related to the number of oxygens in the chemical structure.

6-MITC has many functions, including neuroprotection [18], anti-inflammation [19], anti-pulmonary metastasis [1], chemoprevention [2,20], and cancer prevention [3,21,22]. The mechanisms of 6-MITC inhibit tumor growth through the disruption of mitochondrial function or the inhibition of the NF-κB pathway by abrogating the PI3K/AKT pathway to induce apoptosis [22,23] and sensitizing the anti-tumor agent to augment cell killing [17]. During the cell cycle, the G2/M phase is more sensitive to radiation than other cell cycle phases and more sensitive in the mitotic phase compared to the G2 phase. Our data demonstrated that 6-MITC and I7557 inhibited growth and cancer stem cell-related signaling pathway of human pancreatic cells. We demonstrated that cells treated with a radiosensitizing drug induced G2/M arrest gradually and that it was sustained for 24 h. However, the phosphorylated histone H3 elevated rapidly within a short period of time and peaked after 4 hours duration [24]. In this study, the inhibition of all three compounds against cell growth was evidenced by the induced mitotic arrest or hypoploid. We expressed phosphorylated histone H3 data at 6 h and the cell cycle at 24 h. Our data showed that 6-MITC and I7557 predominately accumulated phosphorylated histone H3, a hallmarker protein of mitotic arrest. The molecules associated with mitotic arrest were phosphorylated forms of CHK1 and Cdc25c. The actual mechanisms of 6-MITC and its chemical derivatives, I7447 and I7557, may be involved in multiple signaling pathways. The evidence suggested by this study warrant further investigation for the development of novel agents against oral cancer.

Anti-cancer agents extracted from natural products have been extensively explored globally. Among these products, naturally occurring compounds isolated from food products are of particular interest due to their relatively safe profile. Wasabi is a pungent spice widely used in food, especially in Japanese cuisine. According to our other study, naturally occurring 6-MITC isolated from wasabi exhibits moderate anti-cancer activity [21]. In this study, we found 6-MITC and its synthetic derivatives possessed stronger activity against human oral cancer cells with low IC_50_ values. Intriguingly, we found that the deletion of oxygen atoms in 6-MITC caused more apoptosis and, vice versa, with the addition of oxygen atoms, resulting in more mitosis in oral cancer SAS cells. This finding implies that the number of oxygen atoms could be a target for further development of novel anti-cancer agents modulating cellular mitosis and apoptosis.

To date, surgical resection is the first curative or therapeutic option for oral cancer. Radiotherapy or concurrent chemo-radiotherapy (CCRT) is an adjuvant treatment for advanced or postoperative disease in oral cancer patients [6]. Although these modalities can improve overall survival, most oral cancer patients with advanced-stage, disease-receiving RT or CCRT experience treatment-related adverse effects, as well as subsequent deleterious effect on their quality of life. More important is concern about the high recurrence of cancer in local or regional areas among oral cancer patients after RT or CCRT. Developing novel radiosensitizers to enhance radioresponse or overcome radioresistance of tumor cells is a current issue. Several strategies have been developed to enhance radiosensitivity in oral cancer. For instance, the proteasome inhibitor, bortezomib, enhanced radiosensitivity through suppression of autophagy-related TRAF6-NF-κB signaling activation [15]. In another study, the authors reported that the mammalian target of the rapamycin (mTOR) inhibitor, RAD001, combined with irradiation could induce G2/M phase arrest to enhance radiosensitivity in oral squamous cancer cells [14]. In the cell cycle distribution, cancer cells in the mitosis phase are most sensitive to radiotherapy [25]. In our study, we tested the radiosensitizing effects of wasabi compounds and found that 6-MITC and I7557 could sensitize SAS cells to radiation with SER, up to 1.02 and 4.64. This radiosensitizing effect might have resulted from the disturbance of phosphorylated CHK1 and Cdc25c that disrupted the G2/M checkpoint. The radiosensitization mechanism is in accordance with previous results [14]. Taken together with this possible structure-activity relationship, the modification of oxygen numbers on 6-MITC may also help to find new radiosensitizers against oral cancer.

## 4. Materials and Methods

### 4.1. Cell Culture

The human oral cancer cell line, SAS, was purchased from the American Type Culture Collection (ATCC, Manassas, VA, USA). HFW were used as the normal counterparts to human oral squamous cell carcinomas. Human oral cancer cell line, OECM-1, and HFW were kindly provided by Dr. Chung-Ji Liu (Taipei, Taiwan) and Te-Chang Lee (Academia Sinica, Taiwan), respectively. SAS and OECM-1 cell lines are squamous cell carcinomas derived from oral cancer patients. SAS and HFW cells were cultured in DMEM (Corning, Manassas, VA, USA), supplemented with 10% heat-inactivated fetal bovine serum (Hyclone, Logan, UT, USA) at 37 °C in a humidified 5% CO_2_ incubator. OECM-1 cells were cultured in RPMI-1640 (Corning, Manassas, VA, USA), supplemented with 10% heat-inactivated fetal bovine serum and 1% penicillin-streptomycin. For cell passage, a working solution containing 0.25% trypsin, 0.1% EDTA, and 0.05% glucose in Hanks’ balanced salt solution was used. Cells were maintained in exponential growth.

### 4.2. Chemicals

The compounds 6-MITC, I7447, and I7557, were purchased from LKT Laboratories (St. Paul, MN, USA) and were dissolved in DMSO prior to experiments. The chemical structures of these compounds are illustrated in Figure 1.

### 4.3. Cell Viability

Cell viability was assayed by a tetrazolium dye, 3-(4,5-dimethylthiazol-2-yl)-2,5-diphenyltetrazolium bromide (MTT) colorimetric method. Human oral cancer cells and HFW cells were cultured in a 24-well plate at a density of 1 × 10^5^ cells/mL. The cells were administered with various concentrations of 6-MITC, I7447, and I7557 for 24 and 48 h. After incubation, tetrazolium dye was added as an indicator, through the conversion of tetrazolium salts to a colored product, formazan. The formazan concentration was measured by spectrophotometer at 570 nm. The half-maximum inhibitory concentration (IC_50_) values were defined as the drug concentration that inhibited 50% cell growth by setting the viability of untreated cells as 100%.

### 4.4. Morphology

For observation of morphological changes, cells were stained with Liu’s stain (Muto Pure Chemicals Co. Ltd., Bunkyou-Ku, Tokyo, Japan). Liu A solution was added for 45 s, followed by adding Liu B solution for 90 s at room temperature. Afterwards, cells were gently washed and dried. Cell morphology was observed under a light microscope (Olympus BX51, Tokyo, Japan) at a magnification of 400×.

### 4.5. Cell Cycle Analysis

Cells were treated with wasabi compound, 6-MITC, and its derivatives for 24 h. After treatment, cells were harvested and fixed with 70% ethanol at 4 °C for 1 h. They were stained with propidium iodide (PI) solution (PI, 0.5 mg/mL; RNase, 0.1 mg/mL) contained in a Cycletest Plus DNA reagent kit (Becton Dickinson, Lincoln Park, NJ, USA). The DNA histograms were analyzed using a FACScaliber flow cytometer (Becton Dickinson, Lincoln Park, NJ, USA). Data from 10^4^ cells were collected and analyzed using ModFit software (Becton Dickinson, Lincoln Park, NJ, USA) to calculate the proportion of each cell cycle phase.

### 4.6. Detection of Phosphorylated Histone H3

Cells were trypsinized, fixed in 2% paraformaldehyde, permeablized with 1% Triton X-100 (Sigma-Aldrich, St. Louis, MO, USA), and stained with anti-phospho-histone H3 (Ser 10)-fluorescein isothiocyanate (FITC) (Cell Signaling, Danvers, MA, USA) at room temperature for 1 h. Then cells were washed with phosphate-buffered saline (PBS) and resuspended in PBS containing PI and RNase A. Prepared cell samples were subjected to flow cytometer with data analysis by using CellQuest^Pro^ software (Becton Dickinson, Lincoln Park, NJ, USA).

### 4.7. Western Blot Analysis

To extract proteins, whole-cell lysates were prepared from cells. A polyvinylidene fluoride membrane was used and blocked with 5% de-fatted milk and then immunoblotted with primary antibodies, including non-phosphorylated and/or phosphorylated forms of CHK2, Cdc2, cyclin B1 (Cell Signaling, Danvers, MA, USA), Cdc25c (GeneTex, Irvine, CA, USA), phosphorylated forms of Cdc25c (Abcam, Cambridge, UK), CHK1 (MBL International corporation, Woburn, MA, USA), phosphorylated CHK1 (Calbiochem, San Diego, CA, USA), and beta-actin (Millipore, Burlington, MA, USA). After adding horseradish peroxidase-labeled second antibodies (Jackson ImmunoResearch, West Grove, PA, USA), immunoreactive proteins were detected using the enhanced chemiluminescence system (GeneTex, Irvine, CA, USA). The expression of beta-actin was used as an internal control for the evaluation of proteins.

### 4.8. Colony Formation Assay

SAS cells (150 cells) were plated onto culture dishes and allowed to grow in McCoy’s 5A medium, containing 20% fetal calf serum and agarose in a humidified incubator with 5% CO_2_. After 10–14 days, the colonies containing ≥50 cells of each were counted. The surviving fraction was calculated as the mean number of colonies/(cells inoculated × plating efficiency). Control plating efficiency for SAS cells was 60–70%. Survival curves were fitted by using a linear-quadratic model [26]. The SER was calculated as the dose of radiation needed for irradiation alone, divided by the dose needed for the test agent plus irradiation to yield a surviving fraction of 37%.

### 4.9. Statistical Analysis

Data are presented as means ± SEM. One-way ANOVA was used to evaluate the difference between groups, and a post-hoc Dunnett’s test was used for pair-wised comparisons. All significant levels (*p*-values) corresponded to two-sided tests (α = 0.05).

## 5. Conclusions

Wasabi compound, 6-MITC, and its chemical derivatives have different oxygen numbers, exhibit a structure-activity relationship, and possess differential pharmacological effects against human oral cancer cells.
